# The Roles of Riblet and Superhydrophobic Surfaces in Energy Saving Using a Spatial Correlation Analysis

**DOI:** 10.3390/nano13050875

**Published:** 2023-02-26

**Authors:** Chunye Liu, Wene Wang, Xiaotao Hu, Juan Fang, Fulai Liu

**Affiliations:** 1 Key Laboratory of Agricultural Soil and Water Engineering in Arid and Semiarid Areas, Ministry of Education, Northwest A&F University, Xianyang 712100, China; 2 School of Energy and Environmental Engineering, University of Science and Technology Beijing, Beijing 100083, China; 3 Department of Plant and Environmental Sciences, University of Copenhagen, 1353 Copenhagen, Denmark

**Keywords:** superhydrophobicity, micro-riblet surfaces, composite micro-riblets and superhydrophobic surfaces, two-point spatial correlation analysis, energy saving

## Abstract

Riblet and superhydrophobic surfaces are two typical passive control technologies used to save energy. In this study, three microstructured samples—a micro-riblet surface (RS), a superhydrophobic surface (SHS), and a novel composite surface of micro-riblets with superhydrophobicity (RSHS)—were designed to improve the drag reduction rate of water flows. Aspects of the flow fields of microstructured samples, including the average velocity, turbulence intensity, and coherent structures of water flows, were investigated via particle image velocimetry (PIV) technology. A two-point spatial correlation analysis was used to explore the influence of the microstructured surfaces on coherent structures of water flows. Our results showed that the velocity on microstructured surface samples was higher than that on the smooth surface (SS) samples, and the turbulence intensity of water on the microstructured surface samples decreased compared with that on the SS samples. The coherent structures of the water flow on microstructured samples were restricted by length and structural angles. The drag reduction rates of the SHS, RS, and RSHS samples were −8.37 %, −9.67 %, and −17.39 %, respectively. The novel established RSHS demonstrated a superior drag reduction effect and could improve the drag reduction rate of water flows.

## 1. Introduction

Developing methods to improve the drag reduction of water flows, especially those involved in water-based engineering such as those encountered by channels, pipelines, and ships in the water, has been crucial to energy savings, mainly because they can result in less fuel and electricity consumption and less harmful emissions. Riblet and superhydrophobic surfaces, which require no other energy input and present effective drag reduction rates in water, are two typical passive control drag reduction technologies [1,2]. Microstructured surfaces, including riblet and superhydrophobic substrates, increase the velocity of water flows with the same energy loss. Different sizes and structures of microstructured surfaces can lead to additional drag reduction and energy-saving effects.

V-shaped riblets, distributed in the streamwise direction at the micro-scale, could provide a drag reduction rate of 9.9% in water flows [3]. The dimensionless spacing *s*^+^ of riblet tips, ranging from 10 to 20, determines the riblets’ drag reduction performance [4]. Martin and Bhushan [5] observed the best drag reduction effect when the dimensionless spacing s^+^ of a V-shaped riblet was 15. Mamori et al. [6] demonstrated that riblet surfaces inhibited the development of vortices near surfaces. Secondary vortices generated at the tips reduced the uplift of vortices, thus minimizing the burst of turbulence flow. Riblets were found to limit the development of streamwise direction vortices and to obstruct the transfer of turbulent kinetic energy. In a previous study, differential pressure was minimized with micro-riblets in natural gas pipelines, and the minimized value was calculated to be about 8–10% [7]. These results demonstrated that micro-riblets reduced wall friction resistance due to the restriction of water flows, while the drag reduction effect of a single type of micro-riblet surface was limited.

Superhydrophobic surfaces provide significant slippage due to their solid, liquid, and gas interface [8]. This interface eventually reduces the contact area between the water flow and the contact surface, thus reducing the surface friction resistance. In addition, superhydrophobic surfaces with a low surface energy are difficult for water droplets to stick to, thus enhancing the sliding property. The slippage of a superhydrophobic coating reduces the frictional shear stress, which leads to more steady flows, resulting in less turbulence. Wang et al. [9] showed that superhydrophobic surfaces could achieve a drag reduction rate of about 20 % under certain conditions. Some research has shown that superhydrophobic substrates have a calming effect on water flows [10]. However, the streamwise slipping produced by superhydrophobic coatings is conducive to drag reduction, while spanwise slipping is not. Therefore, adopting certain methods to reduce the spanwise slipping of superhydrophobic surfaces will help to improve the drag reduction rate. In addition, the nanostructures of superhydrophobic coatings are easily destroyed by the passage of time and changing environments [11,12]. Methods to protect these surface nanostructures should be further explored.

Research on composite micro-riblets and superhydrophobic surfaces is necessary. Kumar et al. [13] stated that using composite microstructured surfaces is one of the best methods for improving drag reduction rates. A superhydrophobic coating can be used to reduce the contact area between water flow and riblets, and streamwise riblets can be expected to limit the spanwise slipping of water, thus reducing the surface friction resistance and increasing the flow velocity near the surface. Additionally, the velocity of water in the grooves of these micro-riblets is low; this property reduces the scouring of water on the superhydrophobic surface and enhances the durability of the superhydrophobic coating. Therefore, micro-riblets protect the nanostructures of superhydrophobic surfaces. 

Spatial correlation analysis has been applied to large-scale researches, such as traffic prediction and environmental pollution [14,15], as well as small-scale projects, such as spectral spatial correlation, microstructure dataset analysis and other topics [16,17]. However, spatial correlation analysis is rarely used in the study of the coherent structures of water flows on microstructured surfaces. The original research methods used to study coherent flow structures mainly focused on proper orthogonal decomposition (POD), finite-time Lyapunov exporters (FTLEs), and large eddy simulations (LESs) [18,19,20]. Hence, using spatial correlation analysis to explore coherent structures of water flows on microstructured surfaces could aid in understanding of the drag reduction mechanism.

Since each passive control drag reduction technology (riblet and superhydrophobic surfaces) has some limitations, improving the drag reduction effects of riblet and superhydrophobic substrates in water flows is worthy of further discussion. Composite micro-riblet and superhydrophobic surfaces have the potential to fix the disadvantages of the two surfaces used alone [21,22]. Hence, in this paper, we established a novel composite surface of micro-riblets with superhydrophobicity to further improve drag reduction rates by using the advantages of both types of surfaces. In addition, the application of spatial correlation analysis to coherent structures has mainly focused on smooth surfaces [23,24], with little use for microstructured surfaces. Ultimately, the study of the composite micro-riblet and superhydrophobic surfaces can be used to improve drag reduction rates and save fuel and electricity.

## 2. Experimental Layouts

### 2.1. Experimental Materials

**Figure 1:**
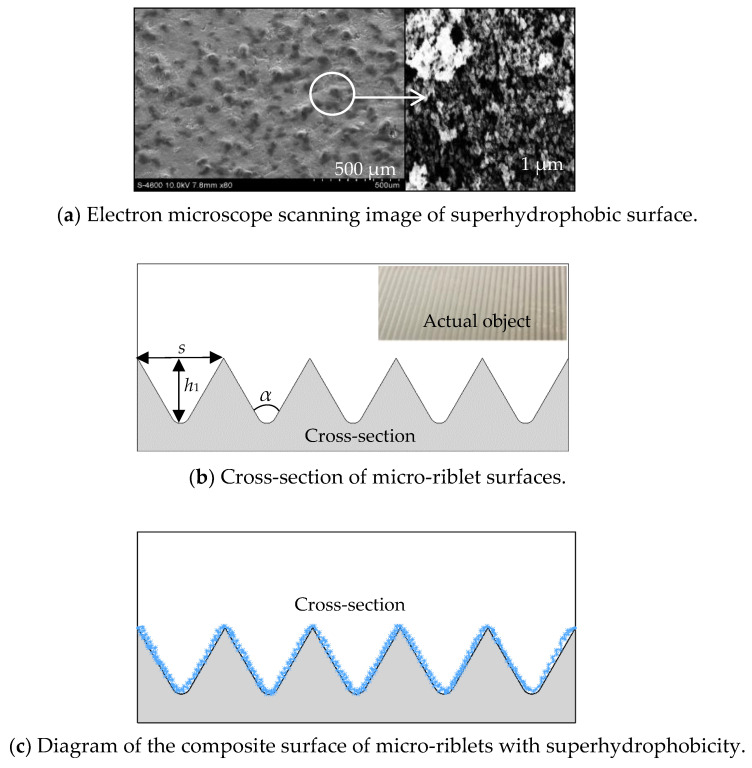
Schematic diagram of microstructured surfaces.

**Figure 2:**
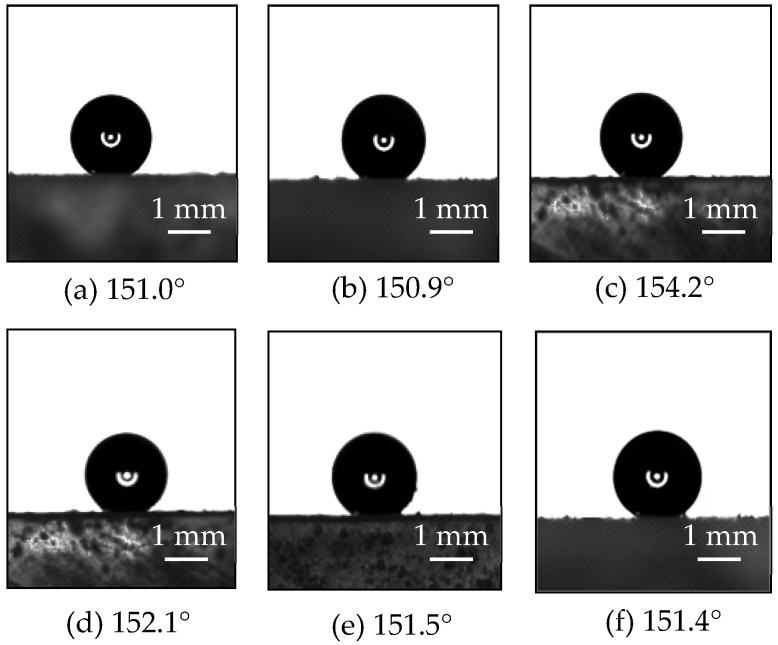
Contact angles of water droplets at different positions on superhydrophobic surfaces.

A superhydrophobic surface (SHS), a micro-riblet surface (RS), and a composite surface of micro-riblets with superhydrophobicity (RSHS) were fabricated using the etching and spraying etching method on acrylic plates; their structures are shown in Figure 1. A smooth surface (SS) served as a control. The SHS was sprayed onto an acrylic plate with never-wet superhydrophobic spray (Rust-oleum, Vernon Hills, Illinois, USA) composed of micro–nano-scale structures. To increase the durability of the coating, the face of the plates was roughened with sandpaper, washed with water, and then washed with alcohol. The distance between the spray and the surface was kept the same, and a method of spraying first horizontally and then vertically was adopted to ensure the same spray thickness while conducting uniform spraying. The structures of the SHS were observed with a scanning electron microscope (Hitachi, Tokyo, Japan). Several different positions of each SHS sample were observed. It was found that the shapes and sizes of the microstructures at different positions were similar to the papillary structures on the surface of lotus leaves, as shown in Figure 1a. The minimum contact angle of the SHS was 150.9°, as measured with a JY-PHB contact angle meter with an accuracy of 0.1° (Jinhe Instrument Manufacturing Co., Ltd., Chengde, China). The contact angles of the SHS at different positions are shown in Figure 2. It should be noted that these are conservative estimates of the contact angles of the superhydrophobic surface because the experiment used water droplets with a volume of 2 μL. When 5 μL of water drops were used, the water drops fell directly from the contact surface. This is why the contact angles measured by our experiments were smaller than those provided by the manufacturer (160–170°). The V-shaped riblet surfaces were fabricated with a V600 CNC machine tool (Dahe CNC Machine Co., Ltd., Yinchuan, China; positioning accuracy of ±0.005/300 mm) at a depth *h*_1_ of 0.8 mm and an angle *α* of 60° [3,5], as shown in Figure 1b. To make the composite micro-riblet and superhydrophobic surfaces, we first constructed micro-riblets on a plate and then sprayed a superhydrophobic layer onto the micro-riblets. A schematic diagram of the composite surface is shown in Figure 1c.

### 2.2. Experimental Devices

**Figure 3:**
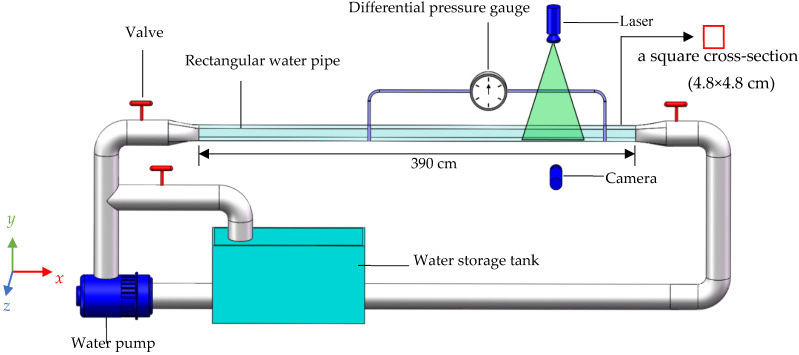
Diagram of experimental circulation pipes.

A water circulation pipe system, including a circulation pipe system, a differential pressure gauge, and a two-dimensional PIV system, was designed for these experiments; see Figure 3. The circulation pipe system was composed of a water storage tank, a water pump, three valves, a rectangular water pipeline with a shrinking section at the head, and a return water pipe. The rectangular pipeline was composed of transparent acrylic with a length of 390 cm in the streamwise direction and a square cross-section (width × height: 4.8 × 4.8 cm). The test section was located 260 cm away from the head of the pipe, and the lengths of the test section and the tailwater section were 60 cm and 70 cm, respectively. The test section was placed away from the head of the pipe to enable the transition of the boundary layer into a fully developed turbulent boundary layer. The test section could be disassembled, and the bottom of the test section was provided with a groove. The microstructured surface was laid into the groove, and the test riblet surface was placed in the streamwise direction of water flows.

The PIV system used in the experiments (Cube World Co., Ltd., Beijing, China) was mainly composed of four parts: a camera (SM-CCDB5M16), double-pulse laser (Vlite-200), synchronization controller, and data analysis system. The laser parameters were as follows: the laser wavelength, energy, and thickness were 532 nm, 200 mJ, and 1 mm, respectively. The maximum shooting frequency was 15 Hz, and the pulse width was ≤8 ns. The particle image resolution was 2456 pixels × 2058 pixels. The main component of the tracer particle (MV-H0510) was SiO_2_ (with a content of more than 65%), and the particle size ranged from 5 to 10 μm. Tracer particles are hollow glass microbeads with a density close to water and good flow-following properties. It was necessary to ensure that there were enough tracer particles in the near-surface area during the experiment. When the number of tracer particles near the surface was small, tracer particles were appropriately added. During particle image processing, cross-correlation calculations and an iterative algorithm were used concurrently, and the initial interpretation area was 32 pixels × 32 pixels. Based on previous calculations, the size of the interpretation area was reduced from 32 pixels to 16 pixels to 8 pixels. The iterative algorithm was used to improve the signal-to-noise ratio of cross-correlation calculations and the accuracy of calculation results. Additionally, an image bias algorithm was introduced, and the windows overlap was 50%. The Gaussian fitting method was used to calculate the sub-pixel calculation error (that is, the accuracy of the calculation result was ±0.1 pixels). The cross-frame time for shooting ranged from 500 μs to 1200 μs. The view field of the captured images was 120 mm × 48 mm (streamwise direction × normal direction, respectively), and the actual resolution of the captured areas was 0.049 × 0.023 mm/pixel.

### 2.3. Experimental Arrangement

Experiments were carried out to observe the flow fields of microstructured surfaces, namely, the SHS, RS, RSHS, and SS samples. The SS was used as a control. In preliminary tests, flow fields near the SHS, RS, and SS could be clearly observed. However, the flow field near the RSHS could not be clearly observed due to the combination of the 60° inclined plane of the micro-riblets and the rough surface of the superhydrophobic coating, resulting in serious laser reflection on the composite surface. However, the laser was not seriously reflected when the superhydrophobic coating was sprayed onto the plane surface. When the laser passed through the junction of water flows and the solid surface, a reflected noise signal was generated. To prevent the noise signal from affecting the experimental results, black background paper was pasted onto the back and bottom of the test section. Hence, two types of experiments were conducted. First, PIV experiments were mainly used to analyze the distribution and variation of the water flow fields near the SHS, RS, and SS. Second, differential pressure experiments were performed to investigate the drag reduction effects of the four different surfaces (SHS, RS, RSHS, SS).

The Reynolds number Re (Re = *uD*/*ν*, where *u* is the average flow velocity, *D* is the hydraulic diameter of the rectangular tube, and *ν* is the kinematic viscosity) ranged from 5375 to 32250. In this experimental Reynolds number range, the dimensionless spacing *s*^+^ (*s*^+^ =su*/*ν*, where *s* is the riblet spacing, u* is the friction velocity, and *ν* is the kinematic viscosity) of the V-shaped riblets was 10–20; in this range, micro-structured surfaces have good drag reduction effects [4,5]. During the experiment, if a drag increase condition occurred within this Reynolds number range, a detailed analysis of the drag increase condition was not carried out; only a Reynolds number with drag reduction effect was analyzed. Three treatments with Reynolds numbers of 5375 (Re_1_), 10750 (Re_2_) and 16125 (Re_3_) were used as examples for detailed analyses. Experimental plates were horizontally placed at the bottom of the rectangular pipe, and the laser was adjusted so that the laser position was perpendicular to the camera shooting position; see Figure 3. Clear particle images were captured by adjusting the laser intensity and camera focal length. Seven hundred images were captured in each group of treatments, and each treatment took approximately 10 minutes. The cross-correlation calculation method was used to calculate the two frames before and after each shooting to calculate the instantaneous velocity in the water flow field. The average velocity of water can be obtained by time-averaging the collected transient velocity. Micro-Vec software in the PIV system was used to obtain tracer particle images. 

The second type of experiment was used to analyze differential pressure changes of the test section to explore the drag reduction effects of the four different surfaces when water flowed. The differential pressure data at different flow rates were collected three times, and the system ran for 5 minutes each time. The differential pressure values were average during experimental processing.

### 2.4. Experimental Indexes

Based on the obtained transient flow velocity values, the average flow velocity distribution, turbulence intensity stress, drag reduction rates, and spatial correlation of fluctuating velocity were analyzed. The calculation formulas are as follows.

1. Average velocity distribution

The average velocity distribution of the logarithmic layer in the turbulent boundary layer is

(1) $$u^{+}=\frac{1}{\kappa }\ln y^{+}+b$$

where *u*^+^ is the dimensionless velocity and *y*^+^ is the normal dimensionless position. Specifically,

(2) $$u^{+}=\frac{u}{u^{*}},\;y^{+}=\frac{yu^{*}}{\nu }$$

where *κ* is the Karman constant; b is a constant number; u^*^ is the wall friction velocity, m/s; *u* is the average velocity, m/s; *y* is the normal distance, m; *ν* is the kinematic viscosity, m^2^/s.

2. Turbulence intensity

Turbulence intensity is a relative index used to measure the strength of turbulence, which can comprehensively reflect the effects of different microstructured surfaces on water flows. The calculation formula is shown in Formula (3):

(3) $$u_{rms}^{+}=\frac{\sqrt{\bar{u^{\prime 2}}}}{u_{*}},\;v_{rms}^{+}=\frac{\sqrt{\bar{v^{\prime 2}}}}{u_{*}}$$

where $u_{rms}^{+}$ and $v_{rms}^{+}$ are the turbulence intensity in the streamwise and normal directions, respectively, and *u*′ and *v*′ are fluctuating velocities in the streamwise and normal directions, respectively.

3. Two-point spatial correlation of fluctuating velocity

Spatial correlation analysis can be used to represent the relationship between spatial positions [16,18]. The correlation of two-point fluctuating velocities represents a large amount of information about the average spatially coherent structures of turbulence [24,25]. Therefore, comparisons of the two-point spatial correlation of microstructured surfaces and the smooth surface can be used to explore changes in the coherent structures of water flows. In this study, the streamwise fluctuating velocity was measured at two positions, *i* and *j*, and the spatial correlation of the fluctuating velocity can be calculated as follows:

(4) $$\bar{u_{i}^{\prime }u_{j}^{\prime }}=\underset{T\to \infty }{\lim}\frac{1}{T}\int_{-T/2}^{T/2}u_{i}^{\prime }u_{j}^{\prime }dt$$

where *u_i_^′^* and *u_j_^′^* represent the fluctuating velocity; *u_i_^′^* = (*x*, *y_ref_*, *t*) and *u_j_^′^* = (*x* + ∆*x*, *y*, *t*), where *x* is the streamwise position, ∆*x* is the spatial distance between two points of the streamwise direction, *y_ref_* represents the reference normal position selected for relevant calculations, and *t* represents time.

The spatial correlation between two points of fluctuating velocity is as follows:

(5) $$\rho (\Delta {x,\;y;\;y}_{\mathrm{ref}})=\frac{\langle u_{i}^{\prime }\left({x,\;y}_{\mathrm{ref}}\;,\;t\right)u_{j}^{\prime }\left(x+\Delta {x,\;y}_{\mathrm{ref}}\;,\;t\right)\rangle }{\left\|u_{i}^{\prime }\left({x,\;y}_{\mathrm{ref}\;},\;t\right)\|\|u_{j}^{\prime }(x+\Delta {x,\;y}_{\mathrm{ref}}\;,\;t)\right\|}$$

where *ρ* represents the two-point spatial correlation of fluctuating velocity between *x* and *x* + ∆*x*, 〈·〉 represents the inner product, and ‖·‖ represents the two-norm.

4. Fanning coefficient

The Fanning coefficient *C_f_* of a wall can be calculated by measuring the differential pressure of a test section [26], and the formula is as follows:

(6) $$C_{f}=\frac{\tau_{w}}{0.5\rho u^{2}}=\frac{\Delta pHW}{\rho u^{2}\left(H+W\right)L}$$

where *C_f_* is the Fanning coefficient; *τ_w_* is wall frictional shear stress, kg/ms^−2^; *ρ* is the density of water, kg/m^3^; *u* is the average velocity, m/s; *∆p* is the differential pressure of the test section, Pa; and *L*, *W*, and *H* are the length, width, and height, respectively, of the test rectangular pipe, m.

The drag reduction rate *DR* is calculated as

(7) $$DR=\frac{C_{f}-C_{f0}}{C_{f0}}\times 100\%$$

where *C_f_* is the frictional resistance of microstructured surfaces, kg/ms^−2^, and *C_f_*_0_ is the frictional resistance of the smooth surface, kg/ms^−2^.

## 3. Results and Discussion

### 3.1. Average Velocity of the Boundary Layer

**Figure 4:**
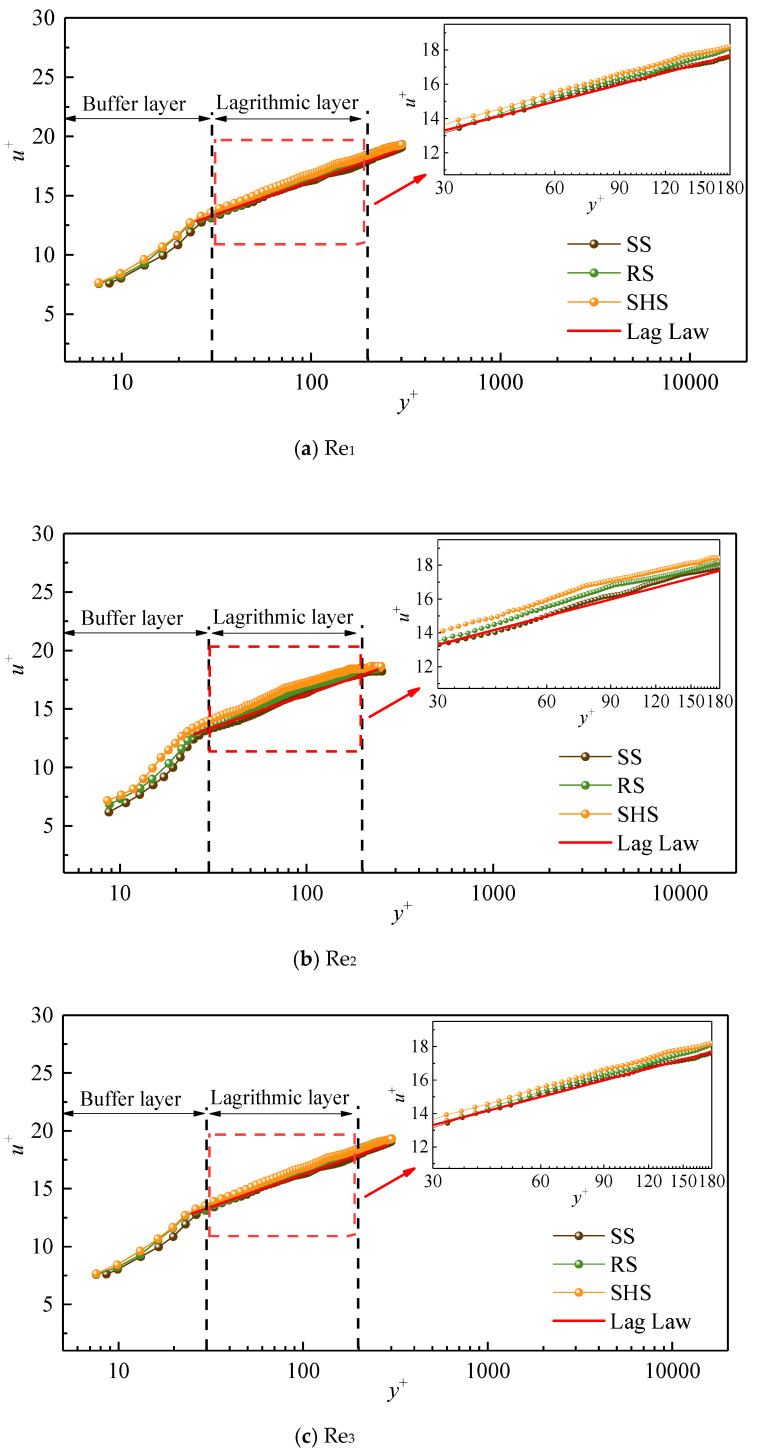
Dimensionless velocity *u*^+^ of microstructured surfaces along the normal distance *y***^+^.**

Figure 4 shows the distribution of the dimensionless average velocity *u*^+^ with the dimensionless normal distance *y*^+^ of the turbulent boundary layer on microstructured surfaces. *u*^+^ and *y*^+^ were calculated according to Formula (2). The value of *u*^+^ increased with the increase in *y*^+^, presenting an evident growth trend, and the velocities of the boundary layer showed an apparent partition phenomenon in different regions of *y*^+^. The turbulent boundary layer was composed of a viscous sublayer (0 < y^+^ < 5), a buffer layer (5 < y^+^ < 30), and a logarithmic layer (30 < y^+^ < 300). Furthermore, the velocity distribution of the logarithmic layer on the smooth surface conformed to the standard log-law distribution, indicating that our results were reliable. Compared with that of the smooth surfaces, the average flow velocity of the microstructured surfaces was increased on the buffer layer. This result was consistent with the research of Wang, who believed that the flow velocity in logarithmic regions would increase on drag reduction surfaces [27]. Considering the enlarged logarithmic view, the velocity distribution of the logarithmic layer was ranked from large to small as SHS, RS, and SS, respectively. Compared with the SS, the flow velocity distribution of the two microstructured surfaces was obviously shifted outward, showing that the average velocity of the microstructured substrates was greater than that of the SS and indicating that microstructures had specific drag reduction effects [28]. Analyzing the reasons for the drag reduction of microstructured surfaces, it has shown that the microstructures of a SHS have a gas–liquid–solid interface, so water droplets on such a surface are prone to relative slippage [29]. Daniello et al. [30] thought that superhydrophobic surfaces had rough micro–nano structures and a low surface energy, and these characteristics reduce the surfaces’ frictional resistance and increase the velocity of their boundary layers. In the scanning electron microscope images of the SHS at 500 μm and 1 μm (Figure 1), it can be seen that micron-scale protrusions with irregular shapes, including fine nano-scale structures, were distributed on the surface, making it easier for water flows to slip along the solid surface at intersecting faces. Additionally, the low energy of the SHS made it difficult for water droplets to adsorb onto the surface. Lee et al. [31] found that the average velocity curve of their studied riblet surface increased because the velocity in the riblet valley was relatively small, resulting in stable, low-velocity streaks, and the tip of the riblets broke streamwise vortices and generated a large number of secondary vortices. In this context, the generation of secondary vortices weakens the turbulence of water flows and reduces the downward sweep of high-speed water flows. Here, the increasing degree of velocity on the two kinds of microstructures gradually decreased with increase in Reynolds number (Re_1_< Re_2_< Re_3_), that is, the outward migration degree of the flow velocity gradually decreased, demonstrating that the drag reduction effect of the microstructured substrates could only be achieved within a specific range of Reynolds number.

### 3.2. Turbulence Intensity on Different Surfaces

**Figure 5:**
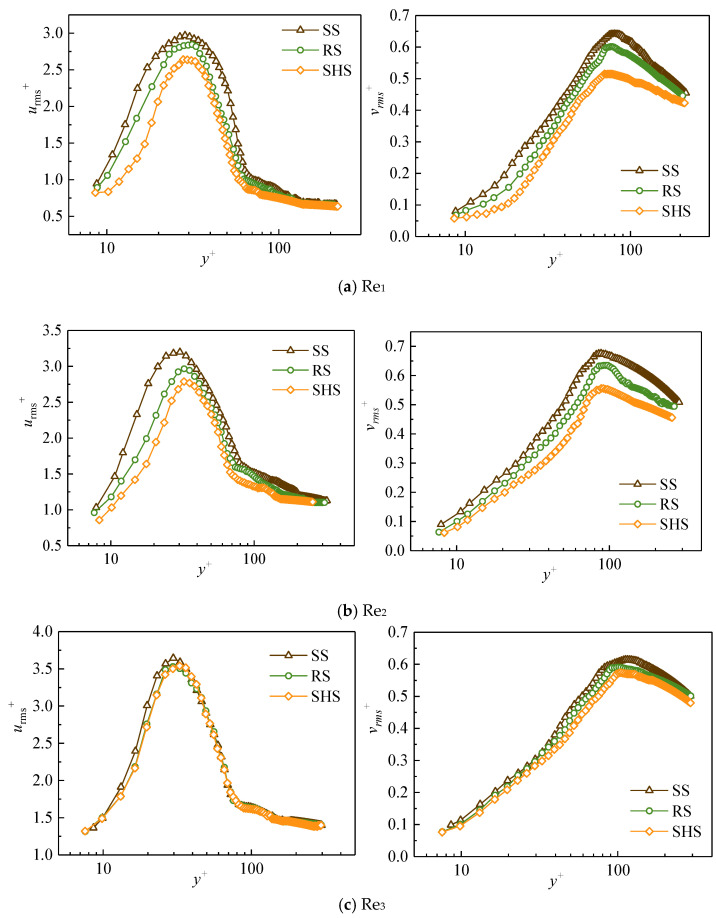
Streamwise and normal turbulence intensity at different Reynolds numbers.

Turbulence intensity can reflect the effects of different micro-surfaces on water flow structures. Figure 5 shows the change of the dimensionless turbulence intensity with the dimensionless normal position *y*^+^ in the streamwise and normal directions; $u_{rms}^{+}$ is the streamwise turbulence intensity, and $v_{rms}^{+}$ is the normal turbulence intensity. The $u_{rms}^{+}$ value at the buffer layer was found to increase with the increase in *y*^+^ until it reached its maximum value, and then $u_{rms}^{+}$ decreased with the rise of *y*^+^ in the logarithmic layer (30 < *y*^+^ < 200). The maximum values of $u_{rms}^{+}$ on the SHS and the RS were less than that of the SS in the buffer layer (5 < *y*^+^ < 30). The buffer layer was the main area where high and low-speed strips existed, and the two kinds of microstructured surfaces showed a greater inhibition effect on the $u_{rms}^{+}$ in the buffer layer. Meanwhile, in the logarithmic law layer, the $u_{rms}^{+}$ on the microstructured surfaces was smaller than that on the smooth surface. Comparing the SHS with the RS, the $u_{rms}^{+}$ on the SHS in the buffer layer was less than that of the RS, indicating that the drag reduction effect of the SHS was better than that of the RS. The $v_{rms}^{+}$ first increased and then decreased with the rise of *y*^+^, and the normal turbulence intensity of the SHS reached its minimum. The maximum values of $v_{rms}^{+}$ from small to large corresponded to the SHS, RS, and SS, respectively. The change in turbulence intensity changes the redistribution mechanism of turbulent kinetic energy, thus changing the generation and transport of water flow structures. Since the pulsation of the normal velocity is closely related to the transmission of turbulent kinetic energy, the existence of riblets weakens the normal pulsation of water flows in turbulent boundary layers, and the intensity of turbulent transmission is also weakened due to the existence of micro-riblets [32]. The reductions in the streamwise and normal turbulence intensities on microstructured surfaces are concrete manifestations of the drag reduction effect.

### 3.3. Spatial Correlation Analysis

There are different conceptions of the coherent structures of water flows due to the diversity of transient flow fields in space. However, all opinions believe that the large-scale coherent structures of water flows are the main factors affecting water flow turbulence [33,34]. To reduce cognitive differences, it is necessary to conduct statistical analyses of transient results in space to objectively evaluate coherent structures of water flows. The coherent structures of water flows mainly consist of various vortex structures. Two-point spatial correlations can reflect a wealth of information about various vortex structures in water flows. Therefore, two-point spatial correlations of fluctuating velocity were used to analyze the vortex structures on the micro-structured surfaces in this study. We investigated the changes in the vortices of water flows on microstructured surfaces by comparing the spatial correlation of the fluctuating velocity between the microstructured surfaces and the SS.

**Figure 6:**
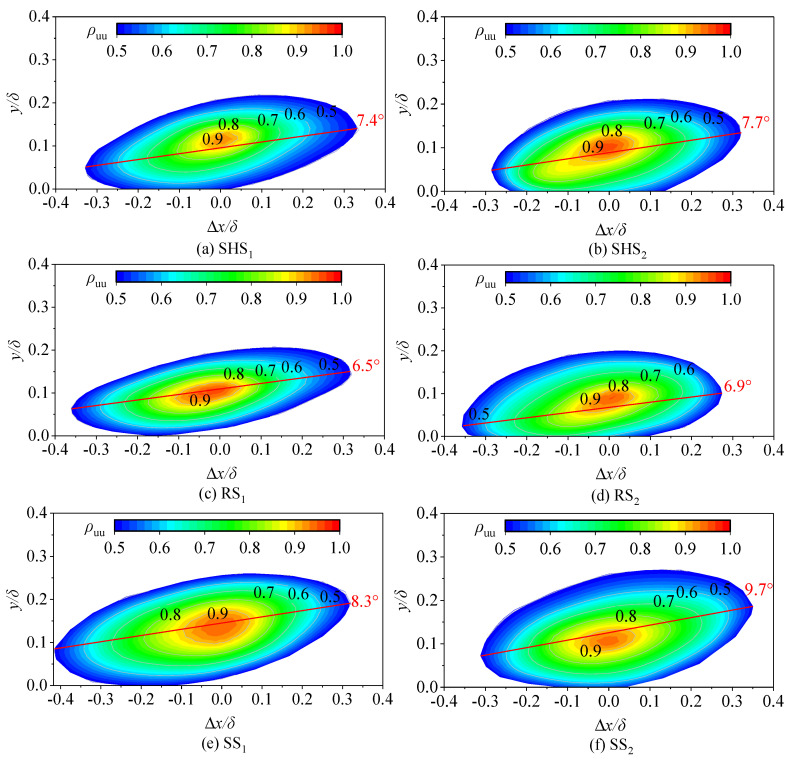
Spatial correlation analysis.

We contrasted the coherent structures of water flows on microstructured surfaces with those of the SS to identify any structural modifications caused by the microstructures. Figure 6 shows the two-point spatial correlation of fluctuating velocity, indicating the spatial characteristics of the coherent structures of the water flows. The spatial correlation analysis was carried out at Re_2_. The main reason was that the water flow velocity on microstructured surfaces was larger when the Reynolds number is Re_2_. At the same normal position *y_ref_* = 0.1*δ*, two reference points, 1 and 2, were set in the streamwise direction. The spatial correlation was centered on a selected reference point, and the results of the spatial correlation calculations were derived from 700 transient particle images to ensure the reliability of the results. In Figure 6, the abscissa ∆*x* is positive on the right of the reference point and negative on the left of the reference point. The ordinate ∆*y* represents the normal height from the surface (both horizontal and normal coordinates are non-dimensional values, ∆*x/δ* and ∆*y/δ*, respectively, where δ is the thickness of the boundary layer). In Figure 6, large-scale coherent structures present an oblique ellipse shape as a whole, with the upstream connection to the surface and the downstream connection far away from the surface due to normal flow movement. It can be seen in the figure that the streamwise direction scale of the structures was much larger than the normal scale. The *ρ*_uu_ value of the center was 1, and it gradually decreased at an interval of 0.1, indicating that the spatial correlation gradually decreased with the increase in the distance from the reference point. Previous studies have shown that spatial correlations can represent the average structure of coherent structures, and a spatial structure with a contour of 0.5 is generally used to describe large-scale coherent structures of water flows [35,36]. In this study, a *ρ*_uu_ value of 0.5 was fitted with the elliptic equation, and the relevant parameters of the ellipse were obtained via the least squares method. L*x* and L*y* are the projections of the long and short axes of the fitted ellipse, respectively, which represent the characteristic length of coherent structures (ordered vortex structures). The structure angle θ is the inclination degree between the major axis of the ellipse and the streamwise (x-axis) direction when the *ρ*_uu_ value is 0.5, representing the deviation between coherent structures and the streamwise direction. The changes of vortex structures in the streamwise and normal directions of the SHS, RS, and SS were quantitatively analyzed via the analysis of their structural parameters. Marusic [36] pointed out that the characteristic length of *ρ*_uu_ corresponds to the distribution characteristics of coherent structures in the streamwise direction and that the influence of the microstructured surface on water flow structures can be reflected through two-point correlation calculations. Chen et al. [37] analyzed the two-point spatial correlation between velocity and vorticity, and they calculated the spanwise spacing, streamwise length, and structure angle of vortices and streaks on a smooth surface. Farano et al. [38] reported that coherent structures developed from vortices of turbulent flows, which reached the outer region of the boundary layer along with streaks, thus proving the importance of vortex structures at spatial scales. Table 1 shows the spatial structural parameters of the coherent structures of water flows on different surfaces studied here. Table 1 shows that the characteristic length L*x* and L*y* of the SHS and RS decreased compared with those of the smooth surface, and the reduction rates are represented by R_L*x*_ and R_L*y*_, respectively. The R_L*x*_ and R_L*y*_ of the SHS decreased by 8.16% and 9.75% while that of the RS decreased by 4.53% and 7.18%. The decrease of R_L*x*_ and R_L*y*_ indicated that these two kinds of microstructured surfaces had particular inhibition effects on the development of streamwise vortex structures. Wu and Christensen [25] maintained that irregular rough surfaces resulted in shorter characteristic lengths in the streamwise direction. In our research, the R_L*x*_ of the SHS was larger than that of the RS, presenting a more significant inhibition effect in the streamwise direction. However, the R_L*y*_ of the RS was larger than that of the SHS, indicating that the RS had a more significant impact in the normal direction. Table 1 shows that the structure angles of the SS were 8.3° and 9.7°. Christensen and Wu [35] measured the structure angles of vortex structures on a smooth surface, which ranged from 8.17° to 11.20°. Jiménez [39] found that the structure angle of spatial correlation on a smooth surface was around 10°. The results of our study on the structure angles of the smooth surface were consistent with previous results.

Most previous scholars have used spatial correlation analysis to explore the coherent structures of water flows on smooth surfaces, and few studies have investigated the spatial correlation of water flow on microstructured surfaces. Therefore, in this study, the spatial structures of water flows on the SHS and the RS were analyzed and contrasted with that of the SS to identify any structural modifications resulting from microstructures. As shown in Figure 6 and Table 1, the structure angles of the SHS were 7.4° and 7.7° and those of the RS were 6.5° and 6.9°. As vortex structures are generated from a surface, they gradually rise in the normal direction over time, resulting in a slightly higher normal position downstream compare to upstream, forming an upward structure angle [40]. Here, compared with that of the SS, the structure angles of the two kinds of microstructured surfaces was reduced. Among them, the RS had the smallest structure angle, and the coherent structures of the RS demonstrated the best effect in reducing the turbulent flow in the normal direction. Stable, low-speed streaks were produced on the surface of the micro-riblets, inhibiting the normal flow turbulence to some extent. Nugroho et al. [28] reported that the interaction of large-scale coherent structures on a surface resulted in significant changes in water flow. Zhang et al. [41] maintained that interface slipping was the main reason for changing water flow structures on superhydrophobic surfaces. According to our analysis, the effects of the SHS and the RS on water flows were different. The SHS had a better inhibition effect on the coherent structures of the water flows in the streamwise direction, while the RS had a better inhibition effect on the water flows in the normal direction. Whether the drag reduction effect of the composite surface would be enhanced if the two surfaces were combined was worth further investigation.

The positive and negative signs in the table represent increases and decreases, respectively.

### 3.4. Differential Pressure Analysis

**Figure 7:**
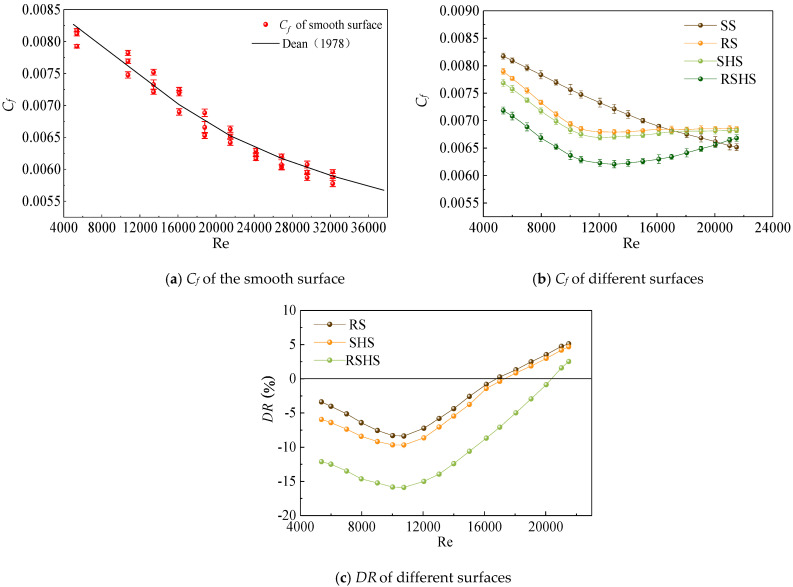
Fanning friction coefficient C*_f_* and drag reduction rate *DR* values.

To analyze the effects of the SHS, RS, and RSHS on water flows, differential pipeline pressures were measured. If the microstructured surfaces inhibited the turbulent flow, the flow velocity near the surface decreased, resulting in a decrease in the differential pressure. The relationship between the flow velocity and the differential pressure was measured, and the Fanning friction coefficient was calculated with formula (6). To verify the reliability of the experimental results, the Fanning friction coefficient on the smooth surface was calculated and compared with the results of Dean [42], as shown in Figure 7. According to Figure 7a, the results measured in this experiment were similar to those reported by Dean, where the maximum deviation was less than 2.9 %. The Fanning friction coefficients of the three microstructured surfaces and the smooth surfaces were compared. Figure 7b shows the results of the comparison. The Fanning friction coefficients of the three microstructured surfaces (SHS, RS, and RSHS) were all smaller than those of the smooth surface, indicating that the microstructured surfaces had specific inhibitory effects on the water flows. The novel established composite surface of micro-riblets with superhydrophobicity (RSHS) had the smallest Fanning friction coefficient and the most potent inhibition effect on water flows. Figure 7c shows that the maximum drag reduction rates of the SHS, RS, and RSHS were −8.37 %, −9.67 %, and −17.39 %, respectively, indicating that the RSHS showed a superior drag reduction effect compared with single microstructured surfaces. To enhance the drag reduction effect, Kim et al. [21] studied a metallic exterior with highly ordered hierarchical structures. They found that the maximum drag reduction rate of metallic hierarchical structures in turbulence was almost 36%. The main reason for the difference between our results and Kim’s results is that the materials used for microstructured surfaces were different, and the various properties of materials result in different drag reduction effects. Rowin et al. [22] reported that when riblets with an *s*^+^ value of 34.6 were combined with a superhydrophobic coating, the composite surface could be changed from the drag-increasing state to the drag-reducing state, and the maximum drag-reducing rate could be increased by 10.2%. In our results, the best drag reduction effect was obtained when the Reynolds number was 10750, as shown in Figure 7c. The *s*^+^ corresponding to the optimal drag reduction effect of the composite surface was 10.7. Previous studies have proved that *s*^+^ had drag reduction effects when *s*^+^ was between 10 and 20 [4,5]. Our results are consistent with previous studies.

## 4. Conclusions

In this study, a novel composite surface of micro-riblets with superhydrophobicity was built to improve the drag reduction rate of microstructured surfaces and realize energy saving. Our results showed that the water flow velocities on two microstructured surfaces (SHS and RS) were larger than that on the smooth surface. The decrease in turbulence intensity on microstructured surfaces indicated that microstructured surfaces could reduce turbulent flow. The main finding of this paper was that the spatially coherent structures of water flows on microstructured surfaces were significantly different from those on the smooth surface. The characteristic length L*x* and L*y* of coherent structures on microstructured surfaces decreased compared with those on the SS. The structural angle ranges of the coherent structures on the SS, SHS, and RS were 8.3° to 9.7°, 7.4° to 7.7°, and 6.5° to 6.9°, respectively. The maximum drag reduction rates of the SHS, RS, and RSHS were −8.37 %, −9.67 %, and −17.39 %, respectively. The novel established surface not only improved the drag reduction rate but also compensated for the shortcomings of a single microstructured substrate and promoted energy savings and emission reductions.

## Figures and Tables

**Table 1:** Characteristic parameters of spatial correlation analysis.

Serial Number	Position on Surfaces	L*x*	L*y*	Angle/°	R_L*x*_/_%_	R_L*y*_/_%_
(a)	SHS_1_	0.666	0.214	7.4	−9.75	−17.69
(b)	SHS_2_	0.608	0.212	7.7	−8.16	−21.77
(c)	RS_1_	0.685	0.207	6.5	−7.18	−23.61
(d)	RS_2_	0.632	0.200	6.9	−4.53	−18.46
(e)	SS_1_	0.738	0.260	8.3	0.00	0
(f)	SS_2_	0.662	0.271	9.7	0.00	0

## Data Availability

The data presented in this study are available on request from the corresponding author wangwene@nwsuaf.edu.cn.
